# Upregulation of circRNA hsa_circ_0008726 in Pre-eclampsia Inhibits Trophoblast Migration, Invasion, and EMT by Regulating miR-345-3p/RYBP Axis

**DOI:** 10.1007/s43032-021-00804-y

**Published:** 2021-11-29

**Authors:** Chang Shu, Peng Xu, Jun Han, Shumei Han, Jin He

**Affiliations:** 1 Department of Obstetrics and Gynecology, The First Hospital of Jilin University, Jilin University, 71 Xinmin Dajie, Chaoyang District, Changchun, 130021 Jilin China; 2 Department of Sports Medicine, The First Hospital of Jilin University, Jilin University, Changchun, China; 3 Neonatal Department, The First Hospital of Jilin University, Jilin University, Changchun, China; 4 Department of Medical Administration, The First Hospital of Jilin University, Jilin University, 71 Xinmin Dajie, Chaoyang District, Changchun, 130021 Jilin China

**Keywords:** circ_0008726, miR-345-3p/RYBP, Pre-eclampsia

## Abstract

Accumulating evidence shows that impaired spiral artery remodeling, placental dysfunction, and insufficient trophoblast infiltration contribute to the etiology and pathogenesis of pre-eclampsia (PE). circRNAs are a class of endogenous non-coding RNAs implicated in the pathogenesis of many diseases, including PE. This study aims to investigate the role of circRNA hsa_circ_0008726 in regulating the migration and invasion of extravillous trophoblast cells. RNase R assay was performed to confirm that circ_0008726 was a circular transcript. The expression of circ_0008726, RYBP, and miR-345-3p was examined by qRT-PCR. The functional interaction between miR-345-3p and circ_0008726 or RYBP was confirmed using dual-luciferase reporter assay and RNA immunoprecipitation (RIP). Cell migration and invasion ability was analyzed by Transwell assays. Western blot was used for the quantification of RYBP protein level. Circ_0008726 expression was significantly increased in PE placenta tissues as compared with normal placenta tissues. Circ_0008726 was resistant to RNase R digestion and was predominately located in the cytoplasm of HTR-8/SVneo cells. Silencing circ_0008726 promoted cell migration and EMT (epithelial-mesenchymal transition), while circ_0008726 overexpression suppressed these processes. Mechanistically, circ_0008726 sponged miR-345-3p to negatively regulate its expression, and miR-345-3p negatively modulated the expression of RYBP. In PE samples, the expression level of circ_0008726 was negatively correlated with miR-345-3p level, but was positively correlated with RYBP expression. Transfection of miR-345-3p mimic or RYBP knockdown counteracted the effects of circ_0008726 overexpression on cell migration and EMT. Our data demonstrate the upregulation of circ_0008726 in PE placenta, which inhibits the migration, invasion, and EMT of HTR-8/SVneo cells by targeting miR-345-3p/RYBP axis. These data suggest that circ_0008726 could be a potential biomarker and therapeutic target for PE.

## Introduction

In normal pregnancy, trophoblast cells migrate through decidua and myometrium and invade maternal spiral artery to provide blood supply to the fetus. Under the condition of pre-eclampsia (PE), trophoblast cells fail to invade the myometrium, which leads to placental hypoperfusion and reduced spiral artery remodeling [1, 2]. Insufficient invasion of placental extravillous trophoblasts (EVTs) at the maternal–fetal interface is considered as the main trigger for the failure of placental formation and the occurrence of PE. Therefore, understanding the pathophysiological mechanism underlying abnormal trophoblast migration and invasion to arteriovenous vessels could provide therapeutic insights into PE [3–5].

CircRNAs are generated by the post-splicing events of precursor mRNAs and show a covalent closed-loop structure without free 3′ and 5′ ends. For a long time, circular transcripts have been regarded as a by-product of abnormal splicing. However, circRNAs have attracted increasing research interest because of their diverse roles in a wide spectrum of physiological and pathophysiological processes, including the regulation of microRNAs (miRNAs), gene expression, and alternative splicing. Many studies have shown that non-coding RNAs are involved in the pathogenesis of PE. For example, a recent study suggests that circ_0111277 attenuates human trophoblast cell invasion and migration by regulating miR-494/HTRA1/Notch-1 signaling axis in PE [6]. However, the potential roles of other circRNAs in PE have been rarely studied.

A previous microarray analysis (GSE102897 and GSE96984) showed that hsa_circ_0008726 (circ_0008726) was highly expressed in patients with PE [7]. Another study indicated that RING1 and YY1 binding protein (RYBP) overexpression remarkably inhibits the proliferation, migration, and invasion of hepatocellular carcinoma cells [8]. In this study, we first verified that circ_0008726 expression was significantly increased in PE placenta tissues as compared to normal placenta tissues. Then, we investigated the potential role of circ_0008726 in trophoblast cell migration. Mechanistically, circ_0008726 serves as a sponge for miR-345-3p in trophoblast cells to suppress their migration and invasion by modulating RYBP. The high expression level of circ_0008726 in PE patients and its functional role in trophoblast migration indicate that circ_0008726 could be a potential biomarker and therapeutic target for PE.

## Materials and Methods

### Patients and Tissue Samples

Placenta tissues from 30 PE patients and 30 healthy controls were collected. All collected samples were immediately washed with sterile phosphate-buffered saline (PBS) before being snap-frozen in liquid nitrogen and stored at − 80 °C for further RNA and protein extraction. The present study was conducted in accordance with the Helsinki Declaration and approved by the hospital Ethics Committee of the First Hospital of Jilin University, and informed consent was taken from all the patients.

### Cell Lines and Culture

HTR-8/SVneo cells (purchased from the Cell Bank of the Chinese Academy of Sciences, China) were maintained in RPMI-1640 medium (Gibco BRL, New York, USA) supplemented with 10% fetal bovine serum (FBS), 100 mg/mL streptomycin, and 100 U/mL penicillin under standard culture conditions (37 ℃ and 5% CO_2_ incubator).

### RNA Extraction and Quantitative RT-qPCR

Total RNA was isolated from tissues or cells using a miRNeasy Kit (Qiagen, Hilden, Germany) according the manufacturer’s protocols. For miRNA analysis, 1 µg of extracted RNA was used for cDNA synthesis by TaqMan MicroRNA Reverse Transcription Kit (Applied Biosystems, Foster City, CA, USA) and quantitative real-time polymerase chain reaction (qRT-PCR) was performed using TaqMan miRNA assay kit (Applied Biosystems). U6 small nuclear RNA (U6 snRNA) was used as an endogenous control for normalization. For mRNA analysis, cDNA was synthesized using M-MLV reverse transcriptase (Invitrogen) with Oligo (dT) as the primer. The qRT-PCR was performed using a 7900HT Fast Real-Time PCR System (Applied Biosystems). Primer sequences used in the present study were as follows:

circ_0008726: forward, 5′-TTGGCTTCACATTCCGTAA-3′, reverse, 5′-CATCCGACAGCACTTCATA-3′

miR-345-3p: forward, 5′-TAGTCCAGGGCTCGTGATGG-3′, reverse, 5′-GGGTCAGAGAGGCTGTCGA-3′.

RYBP mRNA: forward, 5′- CGCAGACGAAGGGTTTTGG-3′ reverse, 5′- GAATTGATCCGAGGTTTTCTGGT-3′

GAPDH primer: forward, 5′-GTAACCCGTTGAACCCCATT-3′, reverse, 5′-CCATCCAATCGGTAGTAGCG-3′.

U6: forward, 5′-CTCGCTTCGGCAGCACA-3′; reverse, 5′-AACGCTTCACGAATTTGCGT-3′

### Chromatin Fractionation

Cells were washed in PBS, and resuspended in Buffer A (10 mM Hepes pH 7.9, 10 mM KCl, 1.5 mM MgCl_2_, 0.34 M sucrose, 10% glycerol, 5 mM NaF, 1 mM Na_3_VO_4_, 1 mM DTT, and protease inhibitor mixture) containing 0.1% Triton X-100, and incubated on ice for 5 min for permeabilization. The cytosolic fraction was then separated by centrifugation at 4000 rpm for 5 min at 4 °C. The supernatant was discarded, and the nuclei pellet was washed once with Buffer A, and resuspended in Buffer B (3 mM EDTA, 0.2 mM EGTA, 1 mM DTT, protease inhibitor mixture), and incubated for 30 min on ice. The soluble nuclear fraction was separated by centrifugation at 4500 rpm for 5 min. The chromatin fraction pellet was washed with Buffer B and resuspended in 100 μL sample buffer and sonicated for 10 s before analysis.

### RNA Pull-Down Assay with Biotinylated RNA Probe

After being transfected with 50 nM biotinylated miR-345-3p probe for 48 h, cells were washed with PBS after brief vortex and incubated with an RNA pull-down lysis buffer on ice for 10 min. After that, the lysates were precleared by centrifugation. The remaining lysates (about 1500 uL) were incubated with M-280 streptavidin magnetic beads precoated with RNase-free BSA (#EN0531; Thermo Scientific) and yeast tRNA (#AM7119; Invitrogen) at 4 °C for 3 h. Then, the beads were washed sequentially with ice-cold lysis buffer, SDS-Tris low salt buffer (pH 8.0 containing 150 mM sodium chloride [NaCl]), and a high salt buffer (containing 500 mM NaCl). The bound total RNA was purified using Trizol reagent (Invitrogen, 15,596,026) according to the manufacturer’s protocol, and analyzed by qPCR.

### Nuclear and Cytoplasmic Fractioning

For nucleoplasm fraction experiment, the nuclear and cytoplasmic faction was extracted using NE-PER™ Nuclear and Cytoplasmic Extraction Reagents (Thermo Fisher Scientific, 78,833), and the total RNA in each fraction was purified using Trizol reagent (Invitrogen, 15,596,026) according to the manufacturer’s protocol. An equal number of cells were used for total cell lysate RNA extraction, which serves as the total cellular RNA level control for normalization. The extracted RNA was quantified by RT-qPCR.

### Cell Transfection

For circ_0008726 overexpression, the circ_0008726 sequence was cloned into the PLCDH-cir vector (RiboBio, Guangzhou, China) [9, 10]. The empty PLCDH-cir vector was the negative control (NC). Control siRNA, siRNA for circ_0008726, miR-345-3p mimic, miR-345-3p inhibitor, and the NC (miR-NC) were synthesized from Life Technologies (Carlsbad, CA). Transfections of above molecules into the cells were performed by Lipofectamine 3000 (Invitrogen, Carlsbad, CA) following the manufacturer’s protocol. Four microgram plasmid and 200 nM of miR-345-3p mimic, inhibitor, or the NC (miR-NC) were used for transfecting cells in 6-well plate with 80% confluency. Forty-eight hours after transfection, cells were harvested for further experiments.

### Dual Luciferase Reporter Assay

293 T cells are used to examine the effect of miR-345-3p on the luciferase reporter of circ_0008726 or RYBP. As indicated in each experiment, cells are transfected with luciferase reporters (RYBP-WT, RYBP-MUT, circRNA-WT, circRNA-MUT) in the presence of miRNA mimic or miR-NC by Lipofectamine 3000 (Invitrogen). After 48 h, luciferase activity was measured using the dual-luciferase reporter assay system according to the manufacturer’s instructions (Promega, Fitchburg, WI, USA). The firefly luciferase reporter activity was normalized to that of Renilla luciferase activity.

### Cell Migration and Invasion Analysis

For the invasion assay, Matrigel (354,230, BD, USA) was diluted 1:30 (V:V) in cold PBS and coated on the bottom of Transwell chamber (CLS3398, Sigma, Germany). For the migration assay, Matrigel was not used. Cells were trypsinized and then resuspended in serum-free medium. 1 × 10^5^ cells HTR-8/SVneo cells were seeded into upper Transwell inserts (8 uM pore size, Corning, USA) which contained RPMI-1640 medium. The lower chambers were filled with RPMI-1640 medium containing 10% FBS, and the Transwell apparatus was cultured at 37℃ with 5% CO_2_ for 48 h. The cells on the upper surface were removed by a cotton swab, fixed by 4% methanol solution for 10 min, and stained by 0.1% crystal violet for 10 min. The number of stained cells was counted under an inverted fluorescence microscope (IX71, Olympus, Japan).

### Western Blot Assay

HTR-8/SVneo cells were lysed by RIPA lysis buffer (Beyotime Biotechnology, China), and the Pierce BCA Protein Assay kit (Thermo Scientific, USA) was used to measure the protein concentration. Twenty-microgram proteins were separated on 10% SDS-PAGE, and then transferred to polyvinylidene fluoride membranes (PVDF, Bio-Rad Laboratories, Inc., USA), which were then blocked in 5% nonfat milk solution for 2 h. The membrane was further incubated with primary antibodies (anti-p-RYBP antibody, rabbit, 1:2000, ab76315, Abcam) and anti-GAPDH antibody (mouse, 1:2000, ab8245, Abcam) at 4℃ overnight. After three washes with TBST buffer, the membranes were incubated with secondary antibodies: goat anti-rabbit IgG H&L (HRP) (goat, 1:2000, ab205718, Abcam) and goat anti-mouse IgG H&L (HRP) (mouse, 1:2000, ab205719, Abcam) for 1 h at room temperature. The protein bands were developed using Pierce Western Blotting ECL substrate kit (Thermo Fisher Scientific, USA) and visualized on the BandScan 5.0 system (Bio-Rad, Hercules, USA). GAPDH was used as the loading control.

### Statistical Methods

Expression levels of circ_0008726 and miR-345-3p were normalized to internal control using 2^−ΔΔCt^ method in qPCR analysis. Data with non-normal distribution for continuous variables were summarized as medians (along with interquartile ranges, IQR) and statistical significance was analyzed by the Mann–Whitney *U*-test. Spearman’s rank correlation coefficients were used to assess potential correlations between two variables. Difference between two groups were compared by Student’s *t* tests. Difference among multiple groups were compared by one-way ANOVA with Tukey’s post hoc test for pairwise comparison. *P*-value less than 0.05 was considered statistically significant. The statistical analysis was performed using GraphPad Prism 7.0 software.

## Results

Clinical characteristics were not significantly different between the 30 PE patients and 30 healthy controls, except for gestational week, birth weight, and placental weight which were directly linked to the PE (Table 1).

**Table 1:** The characteristics of the 30 PE patients and the 30 healthy controls

Parameter	PE group (*n* = 30)	Control group (*n* = 30)	*P*-value
Maternal age (years), mean (SD)	30.4 (1.7)	30.0 (1.0)	0.202
Gestational week, mean (SD)	36.4 (0.8)	38.5 (0.9)	< 0.0001
BMI (kg/m^2^), mean (SD)	26.5 (2.2)	25.3 (1.5)	0.113
> 25 kg/m^2^, no. (%)	19 (63.3)	11 (36.7)	0.07
Nulliparous, no. (%)	14 (46.7)	11 (36.7)	0.601
Previous miscarriage, no. (%)	10 (33.3)	8 (26.7)	0.779
Previous hypertensive pregnancy, no. (%)	8 (26.7)	3 (10.0)	0.181
Maternal blood type, no. (%)			
A	5 (16.7)	7 (23.3)	0.771
B	6 (20.0)	8 (26.7)	
O	12 (40.0)	9 (30.0)	
AB	7 (23.3)	6 (20.0)	
Rh positive, no. (%)	30 (100)	30 (100)	NA
Delivery < 34 w, no. (%)	8 (26.7)	4 (13.3)	0.358
Delivery ≥ 34 w, < 37 w, no. (%)	17 (56.7)	18 (60.0)	
Delivery ≥ 37w, no. (%)	5 (16.3)	8 (26.7)	
Delivery via cesarean-section	24 (80.0)	16 (53.3)	0.054
Vaginal delivery	6 (20.0)	14 (46.7)	
Early-onset PE, no. (%)	17 (56.7)	NA	NA
Chronic hypertension, no. (%)	5 (16.7)	3 (10.0)	0.706
Birth weight (g), mean (SD)	2685.0 (210.8)	3202.0 (401.8)	< 0.0001
Placental weight (g), mean (SD)	521.0 (11.2)	543.2 (19.1)	< 0.0001

*PE*, pre-eclampsia; *BMI*, body mass index; *SD*, standard deviation; *NA*, not available; *w*, week

^#^PE diagnosis: A urine sample is requested for urine dipstick protein test and the blood pressure was determined at every antenatal appointment. If high blood pressure was detected and the dipstick test result was positive for protein, a blood test was further performed to measures the levels of placental growth factor (PIGF). If your PIGF levels are low (< 100 pg/ml), the case was confirmed as pre-eclampsia

### circ_0008726 Is Upregulated in Placental Tissues from PE Women

**Fig. 1:**
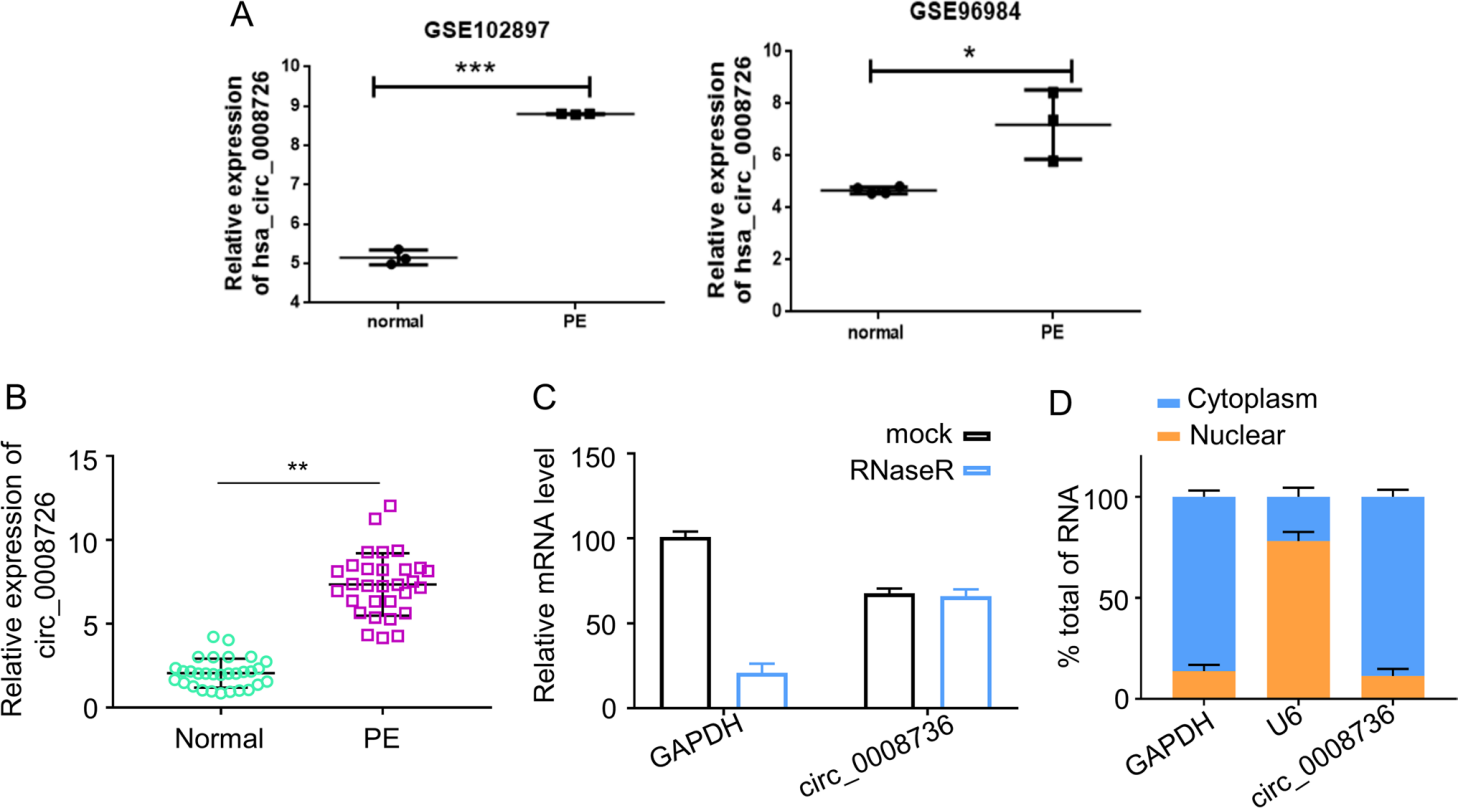
Characterization of circ_0008726. **A** circ_0008726 was highly expressed in the PE tissues of GSE96985 dataset (4 normal tissues and 3 PE tissues) and GSE102897 dataset (3 normal tissues and 3 PE tissues). **B** circ_0008726 levels in PE placentas were compared with those in normal pregnancy placenta by qRT-PCR analysis. **C** qRT-PCR analysis of the levels of circ_0008726 and GAPDH after RNase R digestion. **D** Cell fractioning and qPCR analysis of circ_0008726 localization in HTR-8/SVneo cells. ***p < 0.001, **p < 0.01, *p < 0.05

In order to assess whether Hsa_circ_0008726 (circ_0008726) is related to PE, we retrieved the published microarray datasets surveying the difference in lncRNAs, mRNAs, and circRNAs in PE and normal placenta. circ_0008726 was significantly upregulated in the PE samples in both GSE96985 dataset (4 normal placenta tissues and 3 PE placenta tissues) and GSE102897 dataset (3 normal placenta tissues and 3 PE placenta tissues) (Fig. 1A). We also collected 30 PE placenta tissues and 30 normal placenta tissues, and qPCR analysis showed that circ_0008726 was significantly increased in the PE placenta tissues (*p* < 0.001, Fig. 1B). We next conducted a series of experiments to confirm the circular RNA characteristics of circ_0008726. We applied RNase R, a processive 3′ to 5′ exoribonuclease, to digest total RNA and found that compared with mock group, GAPDH was significantly decreased; however, Circ_0008726 was resistant to RNase R digestion (Fig. 1C), a feature of circular RNA. In addition, cell fractioning and qPCR analysis showed that that circ_0008726 was predominately distributed in the cytoplasm of HTR-8/SVneo cells (Fig. 1D). Collectively, these findings demonstrated that circ_0008726 is a stable and circular transcript.

### Knockdown of circ_0008726 Promotes Trophoblast Migration and Invasion, and Epithelial-Mesenchymal Transition of Trophoblasts

**Fig. 2:**
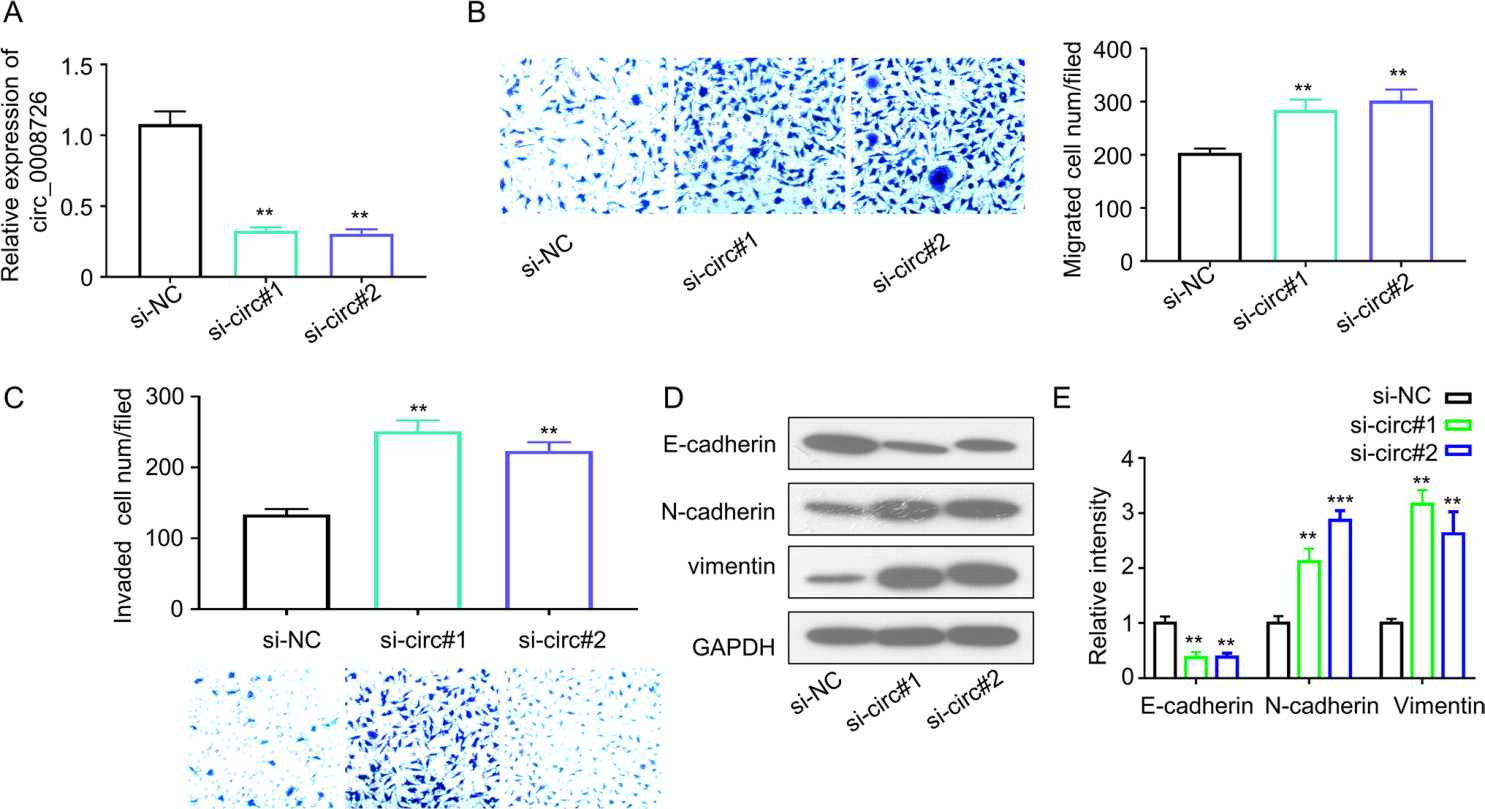
Knockdown of circ_0008726 promotes trophoblast migration and invasion, and EMT of trophoblasts. siRNA knockdown efficiency (**A**), Migration (**B**), and invasion (**C**) capacities of HTR-8/Svneo cells with or without transient transfection of si-circ_0008726 after 48 h. **D** EMT-related markers in HTR-8/SVneo cells determined by Western blot after si-circ_0008726 transfection. **E** Densitometry analysis of protein level in **D**; data normalized the level of GAPDH. ***p < 0.001, **p < 0.01

We next analyzed the functional role of circ_0008726 in trophoblasts by siRNA-mediated silencing. The knockdown efficiency in HTR-8/SVneo cells siRNA was analyzed by qRT-PCR. Transfection of si-circ_0008726#1, si-circ_0008726#2 into HTR-8/SVneo cells could effectively downregulate circ_0008726 (Fig. 2A). Transwell migration and invasion assays demonstrated that silencing circ_0008726 augmented the migratory and invasive capability of HTR-8/SVneo cells (Fig. 2B, C). Epithelial-mesenchymal transition (EMT) plays a crucial role in both normal developmental events (e.g., embryonic development) and pathological conditions (e.g., cancer metastasis), which is characterized of the loss of epithelial feature (E-cadherin) and the upregulation of mesenchymal markers (vimentin and N-cadherin) [11]. EMT is also considered as a major contributor to extravillous trophoblast differentiation and migration [12, 13]. We therefore analyzed the EMT markers by Western blot. In HTR-8/SVneo cells with circ_0008726 knockdown, we observed a diminished level of E-cadherin (epithelial marker) and a heightened expression of vimentin and N-cadherin (mesenchymal markers) (Fig. 2D, E). These data indicate that circ_0008726 negatively regulates EMT process.

### Overexpression of circ_0008726 Suppresses Trophoblast Migration and Invasion, and EMT of Trophoblasts

**Fig. 3:**
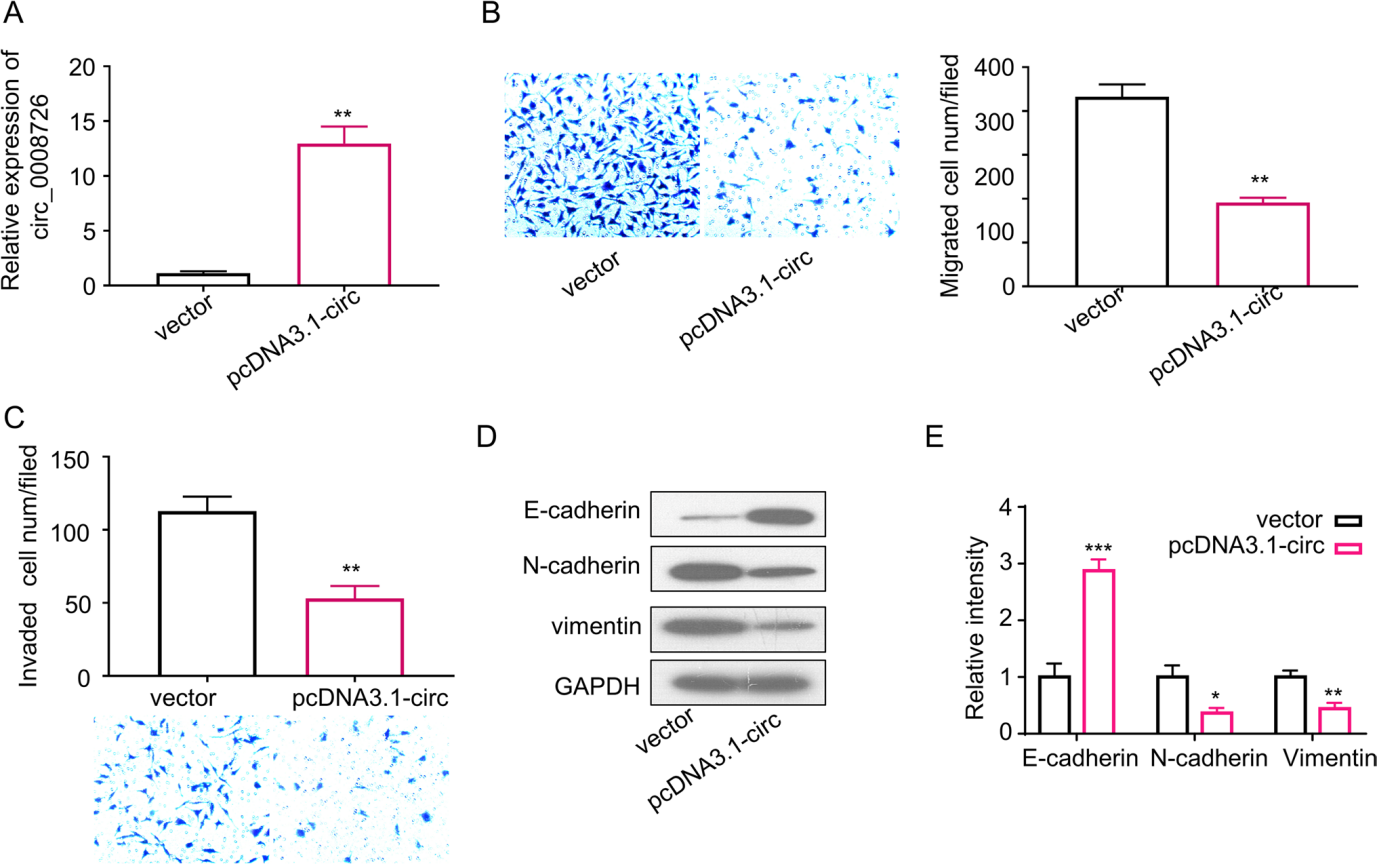
Overexpression of circ_0008726 suppresses trophoblast migration and invasion, and EMT of trophoblasts. Overexpression efficiency (**A**), migration (**B**), and invasion (C) capacities of HTR-8/Svneo cells with or without transient transfection of pcDNA3.1-circ_0008726 after 48 h. **D** EMT-related markers were determined by Western blot in HTR-8/SVneo cells transfected with pcDNA3.1-circ_0008726. **E** Densitometry analysis of protein level in **D**; data normalized the level of GAPDH. **p < 0.01

We next examined the overexpression of circ_0008726 on the migratory features of trophoblasts. The overexpression of circ_0008726 was detected in HTR-8/SVneo cells by qRT-PCR by transfecting the cells with PLCDH-circ_0008726 plasmid (*p* < 0.001, Fig. 3A). Transwell migration and invasion assays demonstrated that circ_0008726 overexpression significantly suppressed the migratory and invasive capability of HTR-8/SVneo cells (Fig. 3B, C). Consistently, Western blot analysis showed that the protein level of E-cadherin increased while vimentin and N-cadherin level decreased in cells overexpressing circ_000872 (Fig. 3D, E). Collectively, these data suggest that circ_0008726 is a negative regulator of migration and EMT in trophoblasts.

### miR-345-3p Is a Target of circ_0008726 in Trophoblasts

**Fig. 4:**
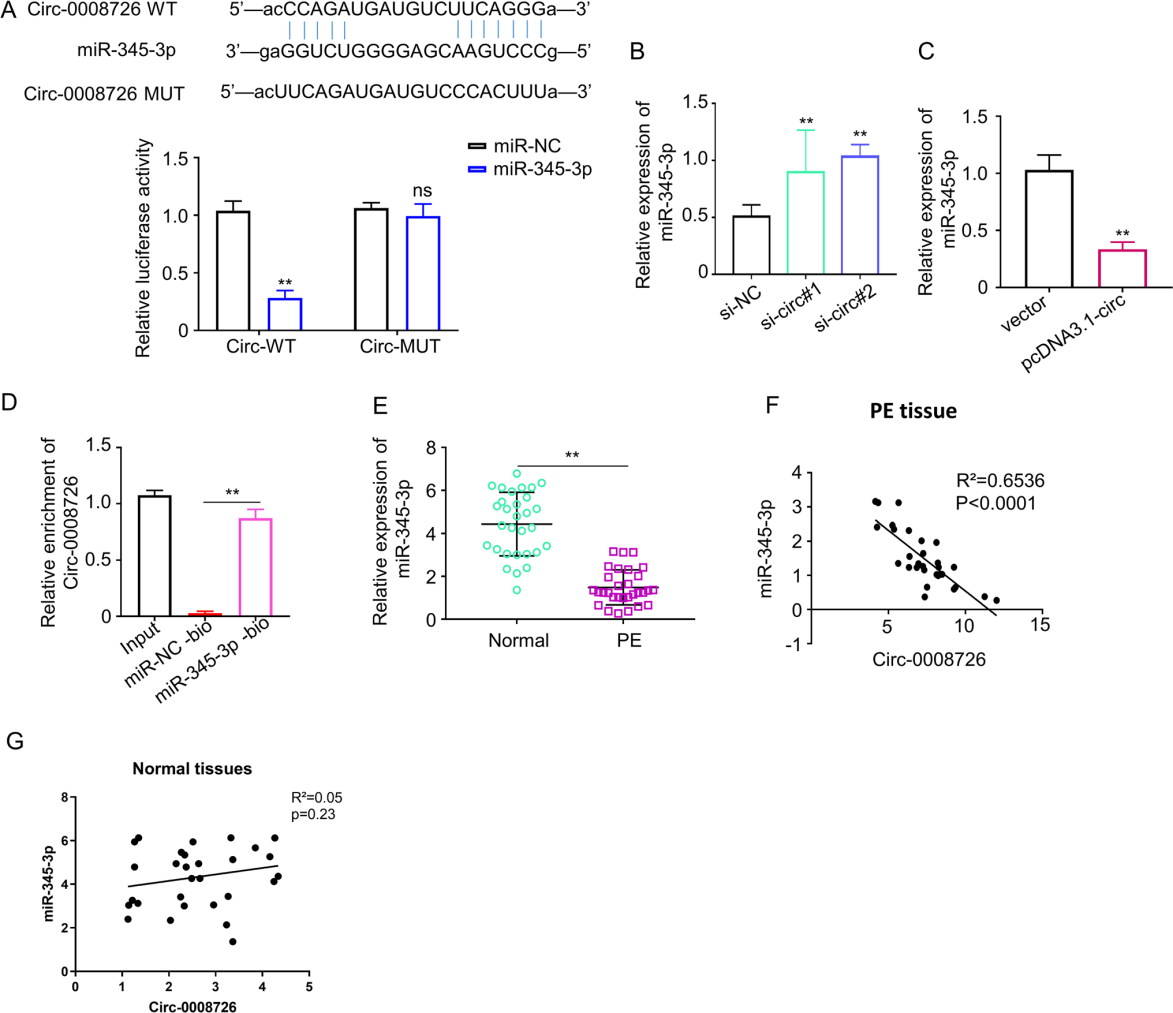
MiR-345-3p is a target of circ_0008726 in trophoblasts. **A** The predicted binding sites of miR-345-3p in circ_0008726 are presented in blue. circ_0008726 wild-type (WT) and mutant (Mut) sites were cloned into luciferase reporter vector and analyzed by dual-luciferase reporter assay. **B**–**C** MiR-345-3p level in HTR8/SVneo cells after the transfection of si-circ_0008726 (**B**) or PLCDH-circ_0008726 (**C**) was examined by qPCR. **D** RNA pull-down analysis using biotinylated miR-345-3p probe or miR-NC probe, and the precipitated RNA was analyzed by qPCR. **E** The expression level of miR-345-3p in PE placental tissues and normal placental tissues was analyzed by qPCR. **F** The correlation between circ_0008726 and miR-345-3p in PE placental tissues. **G** The correlation between circ_0008726 and miR-345-3p in normal tissues. **p < 0.01

We next sought to identify the molecular target of circ_0008726. MiR-345-3p was identified as a potential target of circ_0008726 according to ENCORI online tool (http://starbase.sysu.edu.cn/). To further confirm this result, we performed dual-luciferase reporter assays by constructing wild-type (WT) and mutant (MUT) luciferase reporter vectors of circ_0008726 based on the potential binding site between miR-345-3p and circ_0008726 (Fig. 4A). The luciferase reporters were co-transfected with miR-345-3p mimic or the negative control (miR-NC) into 293 T cells. The WT luciferase reporter activity was significantly inhibited by miR-345-3p mimic, which was not observed in the MUT reporter (Fig. 4A). Moreover, we found a significant increase or decrease of miR-345-3p level when circ_0008726 was silenced or overexpressed in HTR-8/SVneo cells, respectively (Fig. 4B, C). In addition, RNA pull-down experiment demonstrated that miR-345-3p probe significantly enriched circ_0008726 in HTR-8/SVneo cells as compared to miR-NC probe (Fig. 4D). In the PE placenta tissues, the expression level of miR-345-3p was much lower than normal placenta tissues (Fig. 4E), and Spearman’s rank correlation analysis revealed a significant negative correlation between the expression levels of circ_0008726 and miR-345-3p in PE placentas tissues (Fig. 4F). However, no significant correlation was observed in normal placental tissues (Fig. 4G). The above data suggest that miR-345-3p is a functional interactor of circ_0008726 in PE placental tissues.

### *circ_0008726 Promotes RYBP Expression *via* Sponging miR-345-3p*

**Fig. 5:**
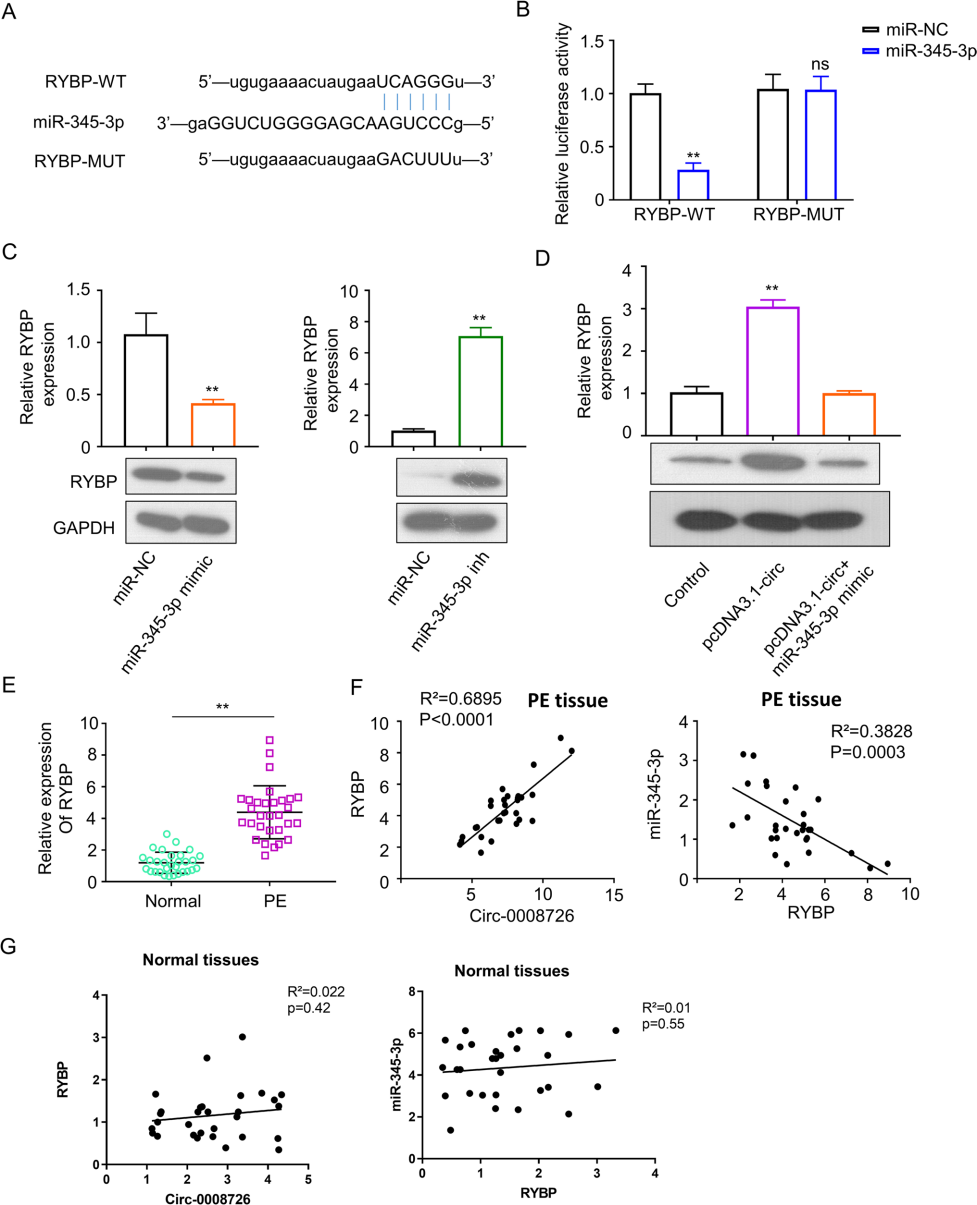
Circ_0008726 promotes RYBP expression via sponging miR-345-3p. **A** The predicted binding sites of miR-494-3p in circ_00087267 were presented in blue. **B** RYBP wild-type (WT) and mutant (Mut) were cloned into luciferase reporter vector and analyzed by dual-luciferase reporter assay. **C** RYBP mRNA and protein level in HTR8/SVneo cells after co-transfection with miR-345-3p mimic or miR-345-3p inhibitor was examined by qPCR (upper) and WB (lower), respectively. **D** RYBP mRNA and protein level in HTR8/SVneo cells after the transfection with pcDNA3.1-circ_0008726 or pcDNA3.1-circ_0008726 + miR-345-3p mimic was examined by qPCR and WB, respectively. **E** The expression level of RYBP mRNA was analyzed in PE placental tissues and normal placental tissues. **F** The correlation between circ_0008726 and RYBP mRNA, and the correlation between miR-345-3p and RYBP mRNA in PE placental tissues. **G** The correlation between circ_0008726 and RYBP mRNA, and the correlation between miR-345-3p and RYBP mRNA in normal placental tissues. **p < 0.01

RYBP was found to a potential target of miR-345-3p based on the target prediction by ENCORI online tool (www.http://starbase.sysu.edu.cn/). To confirm the interaction of miR-345-3p and RYBP mRNA, we performed dual-luciferase reporter experiments by constructing WT or MUT RYBP binding site reporters (Fig. 5A). Compared with miR-NC, the presence of miR-345-3p mimic significantly reduced the luciferase activity of the WT reporter but not in the MUT reporter (Fig. 5B). Knockdown or overexpression of miR-345-3p led to the increase or decrease of RYBP mRNA and protein levels, respectively (Fig. 5C). On the contrary, overexpression of circ_0008726 increased the expression of RYBP mRNA and protein (Fig. 5D). The co-transfection of miR-345-3p mimic suppressed the increase of RYBP expression by circ_0008726 overexpression. Moreover, the expression level of RYBP mRNA in PE placenta tissues was significantly increased when compared with that of normal placenta tissues (Fig. 5E). A significant negative correlation was also observed between RYBP mRNA and miR-345-3p level in PE placenta tissues, while a positive correlation was observed between RYBP and circ_0008726 (Fig. 5F). However, no significant correlation was observed in normal placental tissues (Fig. 5G). Together, these data suggest that RYBP is a target negatively regulated by miR-345-3p.

### *circ_0008726 Inhibits Cell Migration, Invasion, and EMT *via* Targeting miR-345-3p/RYBP Axis in Trophoblasts*

**Fig. 6:**
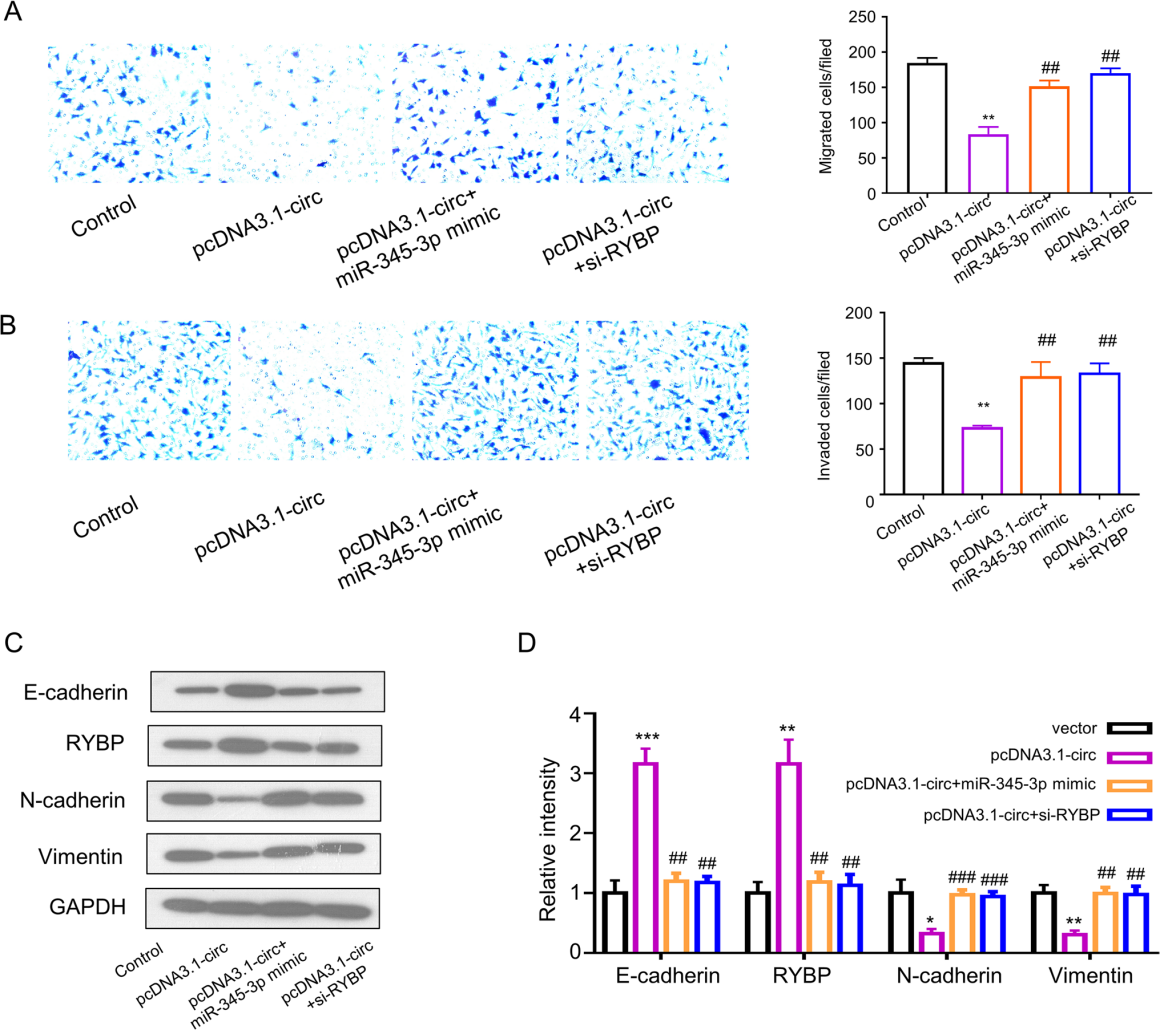
Circ_0008726 inhibits cell migration, invasion, and EMT via targeting miR-345-3p/RYBP axis in trophoblasts. Overexpression of circ_0008726 suppressed trophoblast migration and invasion, and EMT in trophoblasts. Migration (**A**) and invasion (**B**) of HTR-8/Svneo cells after transfection with in pcDNA3.1-circ_0008726, pcDNA3.1-circ_0008726 + miR-345-3p mimic, and pcDNA3.1-circ_0008726 + si-RYBP siRNA. **C** EMT-related marker level was determined by Western blot in HTR-8/SVneo cells transfected with pcDNA3.1-circ_0008726, pcDNA3.1-circ_0008726 + miR-345-3p mimic, and pcDNA3.1-circ_0008726 + si-RYBP siRNA. **D** Densitometry analysis of protein level in **C**; data normalized the level of GAPDH. ***p < 0.001, **p < 0.01, *p < 0.05. (* comparison between controls and PLCDH-circ_0008726; # comparison between co-transfection group with PLCDH-circ_0008726)

We next examined the functional interactions between circ_0008726 and miR-345-3p/RYBP axis. Compared with control group, overexpression of circ_0008726 impaired the cell migration; the inhibition effect of circ_0008726 overexpression was rescued by miR-345 mimic and RYBP silencing (Fig. 6A). Similar results were observed in the Transwell invasion assay (Fig. 6B). Compared to the control group, E-cadherin expression increased and vimentin, N-cadherin expression decreased in circ_0008726 overexpression group. Compared to circ_0008726 overexpression group, E-cadherin expression decreased while vimentin and N-cadherin expression increased in circ_0008726 plasmid and miR-345 mimic co-transfection group. Similar effects were observed when si-RYBP was co-transfected with circ_0008726 plasmid. (Figure C, D). Together, these results demonstrated that circ_0008726 plays a role in suppressing HTR-8/SVneo cell migration and EMT via modulating the miR-345-3p/RYBP axis.

## Discussion

The field of endogenous non-coding RNAs such as circRNAs is attracting more and more research interest [14]. With the development of massively parallel sequencing technology and bioinformatics analysis, a large number of circRNAs have been found to be involved in the occurrence and development of various diseases [15, 16]. Studies have also showed that competitive endogenous RNA (ceRNAs) plays key roles in the pathogenesis of PE [17, 18]. Accumulated evidence indicates that abnormal expression of circRNAs is implicated in PE [6, 19–21]. A number of circRNAs and their targets, such as Circ_0111277/miR-494/HTRA1/Notch-1, circZDHHC20/miR-144/GRHL2, and circPAPPA/miR-384/STAT3, were reported to regulate trophoblast cells migration and invasion [6, 19, 20]. Circ_0063517/miR-31-5p-ETBR axis was also found to regulate the angiogenesis of vascular endothelial cells in PE [21].

**Fig. 7:**
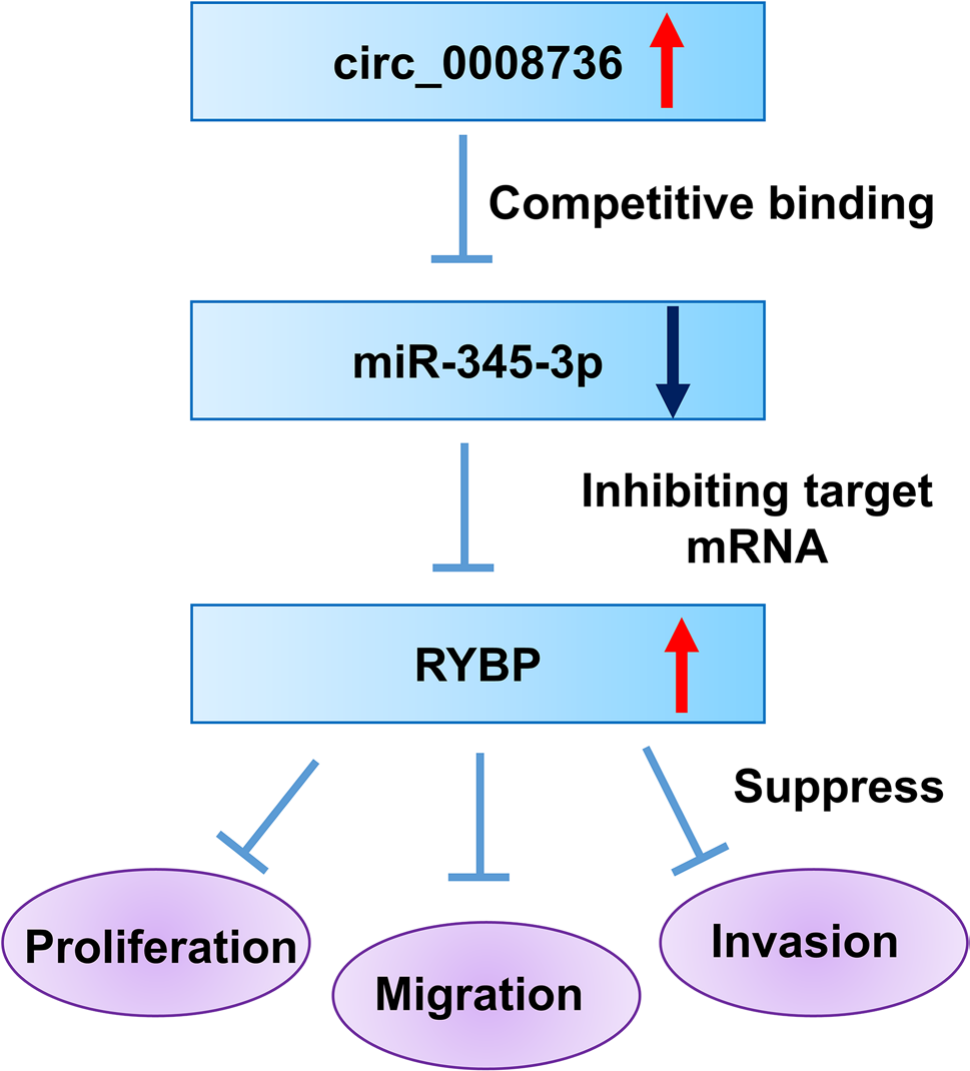
Schematic illustration of circ_0008726/miR-345-3p/RYBP axis in regulating migration and EMT in trophoblast. Overexpression of circ_0008726 in PE trophoblasts suppresses the activity and expression of miR-345-3p, which releases the inhibition on RYBP mRNA. The enhanced RYBP expression contributes to the impaired migration, invasion, and EMT

Consistent with previous studies [19–21], we found that circ_0008726 was upregulated in PE placenta tissues and the overexpression of circ_0008726 plays a functional role to suppress the migration, invasion, and EMT of trophoblasts. Mechanically, we revealed that circ_0008726/miR-345-3p/RYBP axis is involved in the regulation of HTR-8/SVneo migration, invasive, and EMT (Fig. 7). The upregulation of circ_0008726 in PE trophoblasts suppresses miR-345-3p expression level, which releases its inhibition on RYBP mRNA. The enhanced RYBP level contributes to the impaired migration, invasion, and EMT (Fig. 7). MiR-345-3p expression has been reported to be downregulated in diabetes mellitus [22] and diabetic cardiomyopathy [23]. A previous study also found that the expression of miR-345-3p in blood and placental villi of pregnant women with gestational diabetes mellitus is downregulated, which seems to be a downstream target of lncRNA MEG3 [24]. lncRNA MEG3 knockdown promotes cell migration/invasion and reduced cell apoptosis in HTR-8/SVneo cells, while all these effects of lncRNA MEG3 knockdown are abrogated by inhibiting miR-345-3p expression [24]. Although the PE patients are not associated with gestational diabetes mellitus, our results showed that the expression of miR-345-3p in PE placenta tissues was significantly lower than that in normal placental tissues. Together, the previous study and our data suggest that miR-345-3p may be linked to different gestational disorders.

Polycomb repressive complex-1 (PRC1) played a major role in successful progression of stromal cell decidualization [25]. As a polycomb protein, Ring-1 and YY1-binding protein (RYBP) inhibit gene transcription through epigenetic chromatin modification. Pirity MK et al. showed that RYBP is required for early post-implantation and for the central nervous system development [25]. RYBP exhibits distinct upregulation pattern during decidualization with polyploidy and RYBP is predominantly localized in decidual polyploid cells, suggesting that RYBP may participate not only in epithelial differentiation prior to implantation, but also later in terminal differentiation for decidual polyploidy in the post-implantation uterus [26].

Leavey et al. analyzed a total of 330 samples and identified RYBP gene as one of the 3663 differentially expressed genes between PE and non-PE samples [27]. The 3663 genes were then subjected to Gene-Set Enrichment Analysis. Although the expression level of RYBP in tissue was not directly examined further by either PCR or Western blot in their study, clustering analysis in their samples revealed an over-representation of genes involved in the cellular responses to stress pathway (including RYBP) in PE. It would be interesting to investigate the RYBP gene expression level in PE patients on a large scale to further verify our findings. RYBP induces cell cycle arrest and accelerates p53-mediated apoptosis [28]. Infection with adenovirus-RYBP can promote apoptosis and inhibit the proliferation of tumor cells [29]. In accordance with our findings, overexpression of RYBP has been linked with a better prognosis in hepatocellular carcinoma and non-small cell lung cancer (NSCLC) [30, 31]. Overexpression of RYBP in breast cancer cells and lung cancer cell also significantly impaired cell proliferation, migration, and invasion ability [8, 32, 33]. These results are consistent with our findings that RYBP is implicate in regulating the migration of trophoblast cells. However, the molecular mechanism of RYBP in lung cancer metastasis and the relationship between RYBP and EMT is unclear. Overall, our results and previous studies indicate that RYBP seems to play a role in regulating migration in different cells such as cancer cells and trophoblasts.

It is worth mentioning that this study has a few limitations. First, the sample size of PE patients and healthy controls were relatively small. In addition, since pathogenesis of PE is thought to occur in the first trimester, collecting PE patient samples in first semester will be more clinically relevant.

Second, these findings need to be validated in animal models. In addition, other potential downstream targets of circ_0008726 need to be identified through sequencing-based methods.

In summary, our data show that circ_0008726 level is upregulated in PE placental tissues. circ_0008726 seems to function as a sponge for miR-345-3p in trophoblast cells to impair the migration and invasion by regulating RYBP. Our findings indicate a functional role of circ_0008726 in regulating the migration, invasion, and EMT of trophoblasts. However, PE is a complex and clinically heterogeneous disease involving multiple pathophysiological mechanisms. Future study is required to further evaluate the role of circ_0008726 in regulating trophoblast migration in animal model.

## Data Availability

Inquiries of original data can be directed to the corresponding author, and will be provided upon reasonable request.
